# Precision prognostics for the development of complications in diabetes

**DOI:** 10.1007/s00125-022-05731-4

**Published:** 2022-06-21

**Authors:** Catarina Schiborn, Matthias B. Schulze

**Affiliations:** 1 Department of Molecular Epidemiology, German Institute of Human Nutrition Potsdam-Rehbruecke, Nuthetal, Germany; 2 German Center for Diabetes Research (DZD), Neuherberg, Germany; 3 Institute of Nutritional Science, University of Potsdam, Nuthetal, Germany

**Keywords:** Cardiovascular diseases, Complications in diabetes, Macrovascular complications, Microvascular complications, Personalised medicine, Precision medicine, Precision prognostics, Review, Risk prediction, Risk scores

## Abstract

Individuals with diabetes face higher risks for macro- and microvascular complications than their non-diabetic counterparts. The concept of precision medicine in diabetes aims to optimise treatment decisions for individual patients to reduce the risk of major diabetic complications, including cardiovascular outcomes, retinopathy, nephropathy, neuropathy and overall mortality. In this context, prognostic models can be used to estimate an individual’s risk for relevant complications based on individual risk profiles. This review aims to place the concept of prediction modelling into the context of precision prognostics. As opposed to identification of diabetes subsets, the development of prediction models, including the selection of predictors based on their longitudinal association with the outcome of interest and their discriminatory ability, allows estimation of an individual’s absolute risk of complications. As a consequence, such models provide information about potential patient subgroups and their treatment needs. This review provides insight into the methodological issues specifically related to the development and validation of prediction models for diabetes complications. We summarise existing prediction models for macro- and microvascular complications, commonly included predictors, and examples of available validation studies. The review also discusses the potential of non-classical risk markers and omics-based predictors. Finally, it gives insight into the requirements and challenges related to the clinical applications and implementation of developed predictions models to optimise medical decision making.

**Graphical abstract:**

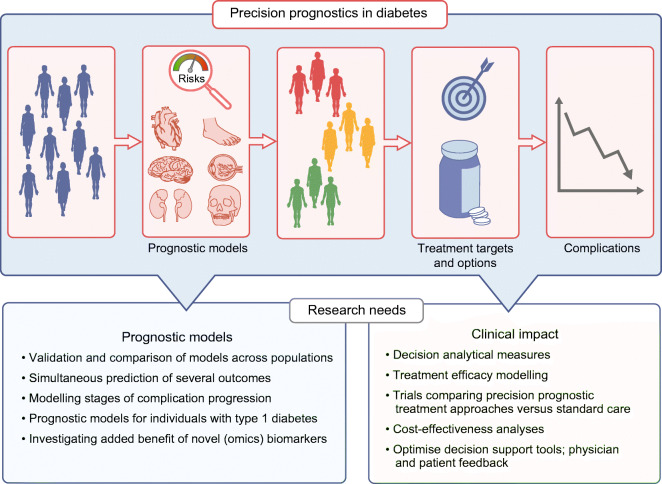

**Supplementary Information:**

The online version contains peer-reviewed but unedited supplementary material including a slideset of the figures for download, which is available to authorised users at 10.1007/s00125-022-05731-4.

## Introduction

Precision medicine in diabetes emphasises tailoring diagnostics or therapeutics to subgroups of populations sharing similar characteristics, thereby minimising error and risk while maximising efficacy [1]. One focus of precision medicine is precision prognostics, which aims to improve the precision and accuracy of predictions of diabetes-related outcomes. CVD (including CHD, cerebrovascular disease and peripheral artery disease) is the leading cause of morbidity and mortality among individuals with diabetes. Diabetes increases the risk of hospitalisation for major CVD events two- to fourfold [2]. According to the Emerging Risk Factor Collaboration, diabetic individuals without prior CVD have a 2.3-fold increased risk of vascular-related death compared with non-diabetic individuals, independent of differences in age, sex, smoking status and BMI [3]. Heart failure risk is similarly increased in individuals with diabetes. Furthermore, microvascular complications (retinopathy, nephropathy, neuropathy) are common in individuals with diabetes and substantially contribute to the burden of comorbidities [4]. Relevant outcomes for precision prognostics in individuals with diabetes include these macro- and microvascular complications and premature death and may also relate to patient-centred outcomes. This review covers the following aspects of precision prognostics in diabetes: (1) methodological approaches for prognostic models; (2) prognostic models for macro- and microvascular complications and overall mortality using routine clinical data; (3) the potential utility of non-classical risk markers; and (4) implementation of precision prognostics in clinical care. Our review focuses on the prediction of diabetes-related macro- and microvascular complications rather than the wider spectrum of diabetes-related comorbidities or patient-centred outcomes.

## Methodological approaches for the development and validation of prognostic models

**Fig. 1:**
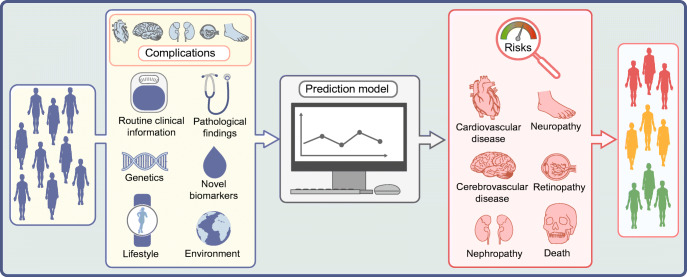
Precision prognostics. Precision prognostics refers to the prognosis of diabetes complications by probabilistic models using information on individual demographic and biological factors (pre-existing complications, routine clinical information, pathological findings, genetics, non-routine [omics-] biomarkers), lifestyle, environment or context. This process allows calculation of an individual’s absolute complication risk, with severity indicated by colour (red, high risk; yellow, medium risk; green, low risk). This figure is available as part of a downloadable slideset

While individuals with diabetes are at higher risk for macro- and microvascular diseases than those without diabetes, the risk is likely to differ substantially from person to person. Diabetes evolves from a variety of pathophysiological constellations, and the presence of other risk factors beyond hyperglycaemia is likely to differ. Precise prognosis of an individual’s likelihood of developing complications would identify those at highest risk, prompting more intensive medical treatment to control risk factors and prevent complications. Precise prognostics allow an individual to be matched to others with a similar complications risk and, through knowledge of treatment efficacy, enable optimal choice of therapy [1]. Precision prognostics refers here to improved precision of prognosis using information on individual biological factors, lifestyle, environment or context [1]. It relates to the development and application of probability-based models, which allow calculation of an individual’s absolute risk for complications based on information from a variety of different risk factors. Prognostic models are based on longitudinal data, with models directly linking information on risk factors to complication events (Fig. 1).

Importantly, precision prognostics differs from attempts to identify subsets of individuals based on physiological variables alone without the use of event information in the process of classification. To illustrate, recent attempts to identify subclasses of diabetes in newly diagnosed individuals [5, 6] allow the matching of a person to a subgroup with a relatively similar phenotype. However, while different event rates for complications might be observable for such subgroups, prognostic models should generally outperform such classification attempts in terms of predictive performance [7].

For prediction models to qualify for implementation into routine care, they should undergo different stages: model development; model evaluation in terms of prognostic performance (ideally including external validation in the target population); translation to clinical decision support; and evaluation of the clinical implementation [8–10]. In the developmental stage, the selection of the study population, predictors, outcomes and the prediction time frame is highly decisive for the subsequent application possibilities, and the choice should fit the intended use of the model (i.e. the study population and setting should mirror the characteristics of the target population for the application). Predictor candidates should be selected based on their predictive ability and for parsimony of the model, yet should also depend on their availably in the envisioned application setting. Furthermore, the prediction time frame should relate to potential interventions to lower risk.

The next crucial step is the evaluation of model performance in terms of discrimination and calibration. Discrimination relates to the model’s ability to differentiate between future cases and non-cases (e.g. by assignment of higher predicted risks to future cases). This is frequently expressed by concordance (C) statistics such as the area under the receiver operating characteristic curve (ROC-AUC) and the *C* index ranging between 0.5 (predicted risk assignment equals chance) and 1.0 (perfect discrimination) [11, 12]. The calibration refers to the agreement between the predicted probability of developing the outcome of interest within a certain time period and the observed outcome frequencies [9, 12]. Assessments of discrimination and calibration are also essential to evaluate prediction increment through additional predictors. However, the C statistic is considered to be insufficiently sensitive to reflect small but clinically meaningful model improvements. Therefore reclassification-based methods such as the net reclassification improvement (NRI) and the integrated discrimination improvement (IDI) have been proposed to complement the evaluation of additional predictors on top of the previously described performance measures [12, 13]. Importantly, to avoid over-optimistic performance estimates from internal validations as a result of overfitting, model performance should be externally validated.

Specifically in the context of diabetes complications, several aspects of the development that may complicate the interpretation, validation and performance assessment need to be taken into account. First, the model performance and its comparability across different studies is highly dependent on the outcome definition. Aggregating multiple complications to one composite, potentially clinically (more) relevant or informative outcome is common practice. CVD models, for example, may predict quite different composite outcomes of myocardial infarction, ischaemic and/or haemorrhagic stroke, heart failure, transient ischaemic attack, angina and other cardiovascular events. The lack of standardised outcome definitions and unavailability of single components of composite endpoints in individual studies hampers the ability to compare different models and model performance across studies. On the other hand, there are also deviations in the diagnostic definitions applied for single endpoints themselves. While there are attempts to standardise cardiovascular event diagnoses and classifications (e.g. by use of the WHO Monitoring Trends and Determinants in Cardiovascular Disease [MONICA] criteria), standardisation appears less common for microvascular complications. Some studies aimed at addressing this issue have derived models for different diagnosis definitions or differently composed endpoints. For instance, the Risk Equations for Complications of Type 2 Diabetes (RECODe) models predicts nephropathy as microalbuminuria, macroalbuminuria, renal failure or end-stage renal disease, doubling of serum creatinine, or >20 ml min^−1^ [1.73 m]^−2^ decrease in eGFR, either alone or in combination [14]. Still, there is a clear need for standardised diagnosis and outcome definitions in prognostic modelling of diabetes complications to allow comparison across different studies.

Second, the pathophysiological interconnection of diabetes-related secondary diseases complicates the prediction of diabetic complications in type 2 diabetes [15–18] and type 1 diabetes [19, 20]. As an example, the development of macrovascular complications is accelerated by the presence of microvascular complications in type 2 diabetes [15]. Beyond that, the development of the interconnected diabetic comorbidities likely underlies a time-dependent gradual process with different stages of progression that could be taken into account to improve risk predictions. When developing prognostic models, different approaches could conceivably address these issues, although each of them comes with specific limitations, as described in Text box 1. 
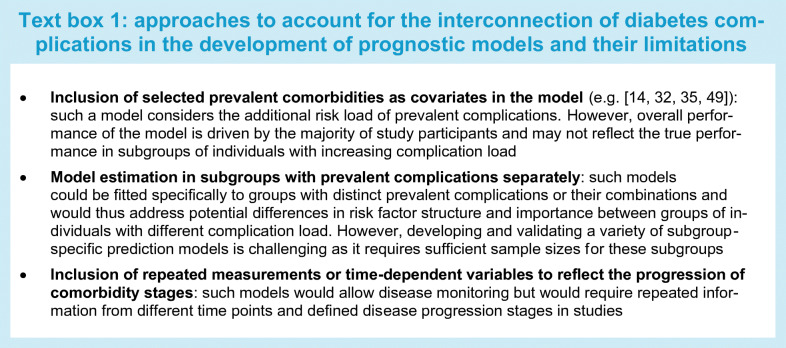


Third, the composition of the study sample used for model development is important. The baseline risk and the estimated weights for individual risk factors incorporated into prognostic models are average-based and depend on the derivation cohort. This potentially conflicts with the concept of precision prevention, as the ‘average’ may not accurately reflect the risk in minorities or subgroups in particularly heterogeneous study samples. One may, for example, argue that separate prognostic models for diabetes complications are needed for the different diabetes clusters [5] rather than a ‘one-model-fits-all’ approach. However, higher homogeneity in terms of individual characteristics in a (sub)sample is related to lower discriminatory ability [21] and may thus complicate the identification of factors that accurately predict events in these subgroups.

## Current status of prognostic models that use ‘classical’ risk factors

**Fig. 2:**
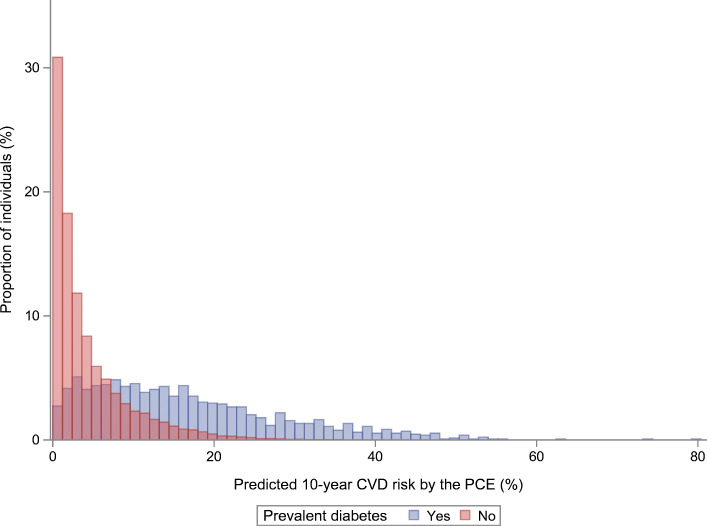
Illustrative example of the distribution of absolute 10 year CVD risk estimated by the Pooled Cohort Equation (PCE) [22] in individuals without and with type 2 diabetes from the European Prospective Investigation into Cancer and Nutrition (EPIC)-Potsdam study (*n* = 25,993) [85]. The distribution of absolute risk of CVD is on average higher in individuals with diabetes compared with individuals without diabetes. While the prognostic model performs well in the full general population, performance within the subgroup of individuals with diabetes may be substantially lower. This figure is available as part of a downloadable slideset

Statistical models for predicting macro- and microvascular complications are widely available. While some models were developed in individuals with diabetes, others (mainly cardiovascular models such as the Pooled Cohort Equation [PCE] [22] and the Framingham risk scores [23, 24]) were initially developed in the general population. Validation efforts suggest that the latter may not provide reliable predictions in individuals with diabetes (e.g. regarding CVD risk) [14, 25–27]. As already mentioned, this might be explained by difficulties in accurately predicting risk in specific population subgroups. This point is illustrated in Fig. 2, which shows the markedly different distribution of predicted CVD risk in individuals with vs without diabetes. As a consequence, CVD prediction models developed for general populations show lower discriminatory ability in individuals with diabetes compared with models specifically developed in populations of individuals with diabetes [28]. Accordingly, this section focuses on models developed in study populations restricted to individuals with diabetes, with an emphasis on type 2 diabetes.

### Risk models for the prediction of macrovascular complications

Among models predicting absolute risk of macrovascular complications [28–30], the majority originate from study samples located in Europe (the UK Prospective Diabetes Study [UKPDS] risk engines and outcomes models 1&2 [31–34], Action in Diabetes and Vascular Disease: Preterax and Diamicron MR Controlled Evaluation [ADVANCE] model [35] and two Swedish National Diabetes Register [NDR] models [36, 37]) or the USA and/or Canada (e.g. RECODe models [14], the Cardiovascular Health Study [CHS] score [38] and Atherosclerosis Risk in Communities [ARIC] model [39]). Three recent meta-analyses pooled the discriminatory measures of selected risk scores with at least two available external validations for different outcome definitions [28–30] (Table 1). They reported pooled C statistics for CVD ranging from 0.66 for the UKPDS risk engine for CHD [34] to 0.70 for the Fremantle risk score [40]. For stroke outcomes [30], the pooled C statistic varied from 0.66 for the UKPDS outcomes model 1 [31] to 0.75 for the Fremantle risk score [40]. In a separate meta-analysis investigating the prediction of cardiovascular death, myocardial infarction and stroke, the RECODe models outperformed other models in terms of pooled C statistic for all three outcomes (cardiovascular death 0.79, myocardial infarction 0.72, stroke 0.71) [29]. However, there were substantial differences in discrimination across individual cohorts used for external validation. For example, the C statistic (95% CI) of the Fremantle risk score ranged between 0.58 (0.50, 0.66) and 0.69 (0.59, 0.79) for the prediction of CVD in the European Prospective Investigation into Cancer and Nutrition (EPIC)-NL, EPIC-Potsdam and the Secondary Manifestations of ARTerial disease cohorts [41] (Table 2). Hence, some scores may be better suited for some specific populations than for others. Direct comparisons of models within populations seems highly informative here. Fewer models have been developed for the prediction of macrovascular complications in type 1 diabetes; such models include the externally validated Steno T1D Risk Engine [42], the Swedish NDR [43] and the Scottish NDR risk score for type 1 diabetes [44].

**Table 1:** Discrimination performance of diabetes-specific cardiovascular risk models in meta-analyses of validation studies [28–30]

Model	Chowdhury et al [28]^a^	Chowdhury et al [30]^b^	Buchan et al [29]^c^		
	CVD	Stroke	CV-related death	MI	Stroke
UKPDS, Stevens et al, 2001 [34]	0.66 (0.60, 0.72)	^d^			
UKPDS, Kothari et al, 2002 [33]	^d^	0.72 (0.68, 0.75)			
UKPDS OM 1, Clarke et al, 2004 [31]		0.66 (0.61, 0.71)	0.70 (0.59, 0.81)		0.70 (0.66, 0.74)
UKPDS OM 2, Hayes et al, 2013 [32]	^e^		0.68 (0.61, 0.75)	0.64 (0.58, 0.70)	0.60 (0.59, 0.61)
ADVANCE, Kengne et al, 2011 [35]	0.69 (0.67, 0.71)	0.67 (0.65, 0.69)			
DCS, Elley et al, 2010 [86]	0.68 (0.66, 0.69)	^f^			
Fremantle, Davis et al, 2010 [40]	0.70 (0.59, 0.81)	0.75 (0.58, 0.92)			
NDR, Cederholm et al, 2008 [36]	0.67 (0.64, 0.71)	^f^			
NDR, Zethelius et al, 2011 [37]	^f^	0.69 (0.63, 0.75)			
CHS, Mukamal et al, 2013 [38]		0.67 (0.67, 0.68)			
RECODe, Basu et al, 2017 [14]	^g^	0.71 (0.67, 0.69)	0.79 (0.75, 0.83)	0.72 (0.70, 0.74)	0.71 (0.68, 0.74)

Discrimination is depicted as pooled C statistic (95% CIs) based on at least two external validations of the according model

^a^Until 12 April 2016

^b^Until 22 April 2019

^c^Until January 2020

^d^Mismatch in outcome definition

^e^Not considered because computer-simulation based

^f^Less than two external validations for the according outcome by the time of systematic search

^g^Score published after systematic search was completed

CHS, Cardiovascular Health Study; MI, myocardial infarction; OM, outcomes model

**Table 2:** Performance comparison of selected models predicting cardiovascular outcomes in individuals with diabetes extracted from external validation studies including at least two statistical models

Cohort	No. of overall participants/cases	Discrimination (C statistic)									
		RECODe [14]	UKPDS CHD [34] or (†) stroke [33]	UKPDS OM2 [32]	CHS [38]	ADVANCE [35]	Fremantle [40]	DCS [86]	ARIC [39]	NDR [36] or (†) [37]	Yang et al CHD [87] or (†) stroke [88]
EPIC-NL [41]	453										
CVD^a^	52				0.54	0.62	0.58	0.63		0.64	
CHD^b^	32		0.61					0.59	0.55		0.63
EPIC-Potsdam [41]	1174										
CVD^c^	41				0.61	0.67	0.68	0.66		0.67	
CHD^d^	23		0.73					0.68	0.69		0.68
SMART [41]	584										
CVD^e^	29				0.68	0.68	0.69	0.67		0.64	
CHD^f^	14		0.66					0.62	0.76		0.61
ADVANCE [27]	7502										
Major CHD^g^	241		0.71								
Any CHD^h^	407		0.66								
Major cerebrovascular^i^	207		†0.62								
Any cerebrovascular^j^	288		†0.61								
Look AHEAD [14]	4760										
ASCVD^k^	462	0.73		0.67							
MI	332	0.71		0.67							
Stroke	157	0.67		0.63							
CHF	210	0.76		0.61							
CVD mortality	106	0.79									
ACCORD [14]	9635										
ASCVD^k^				0.62							
MI	880			0.62							
Stroke	197			0.61							
CHF	454			0.61							
MESA [62]	1555										
ASCVD^k^		0.74		0.60							
MI	92	0.73		0.54							
Stroke	89	0.75		0.60							
CHF	117	0.80		0.57							
CVD death	88	0.81									
JHS [62]	1746										
ASCVD^k^		0.77		0.61							
MI	151	0.74		0.57							
Stroke	142	0.72		0.60							
CHF	161	0.73		0.54							
Scottish NDR [89]	181,399										
CVD^l^	14,081				0.67	0.67	0.67	0.67		†0.66	
Hong Kong health records [90]	678,750										
Cerebrovascular disease^m^	43,215		†0.68	0.65							†0.72
IHD^n^	54,365		0.66	0.65							0.66

^a^AMI, IHD, stroke, sudden death or HF

^b^AMI, IHD

^c^AMI or stroke

^d^AMI

^e^AMI, stroke and vascular mortality

^f^AMI, sudden cardiac death

^g^Death from CHD, sudden death, non-fatal MI

^h^Major CHD, coronary revascularisation and hospitalisation for unstable angina

^i^Death from cerebrovascular events, non-fatal stroke

^j^Stroke, TIA

^k^Non-fatal or fatal MI or stroke

^l^Hospital admission or death from MI, stroke, unstable angina, TIA, peripheral vascular disease and coronary, carotid, or major amputation procedures

^m^Intracranial haemorrhages (e.g., subarachnoid, intracerebral) and occlusion of cerebral arteries

^n^MI, angina pectoris, coronary atherosclerosis and aneurysms

ACCORD, Action to Control Cardiovascular Risk in Diabetes; AHEAD, Action for Health in Diabetes; AMI, acute myocardial infarction; ARIC, Atherosclerosis Risk in Communities; ASCVD, atherosclerotic CVD; CHF, congestive heart failure; CHS, Cardiovascular Health Study; HF, heart failure; IHD, ischaemic heart disease; JHS, Jackson Heart Study; MESA, Multi-Ethnic Study of Atherosclerosis; NL, the Netherlands; OM, outcomes model; SMART, Secondary Manifestations of ARTerial disease cohorts; TIA, transient ischaemic attack

There is considerable overlap regarding the incorporated predictors (see Table 3 for examples), with most models including demographic characteristics such as age, sex (as a covariate or by estimating sex-specific models) and ethnicity, and lifestyle-related variables such as smoking status, disease history, HbA_1c_ or diabetes duration.

**Table 3:**
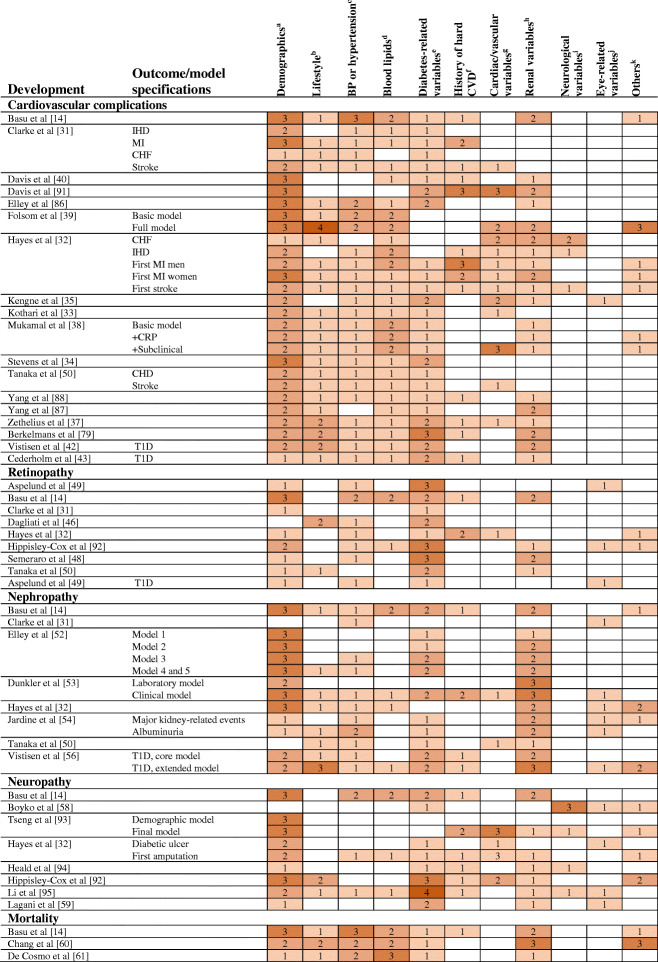
Predictors included in statistical models predicting macro- and microvascular complications in diabetic individuals

Single predictors are aggregated to categories. Colour scheme numbers indicate the numbers of individual predictors included in the corresponding predictor categories. For full table see ESM Table 1

^a^Includes age, sex, ethnicity

^b^Includes smoking status, BMI, waist circumference, waist/hip ratio, physical activity

^c^Includes systolic BP, diastolic BP, hypertension, treated hypertension, BP-lowering drugs, statins, use of diuretics and nitrates, ACE inhibitors

^d^Includes total cholesterol, HDL-cholesterol, total cholesterol/HDL-cholesterol ratio, LDL-chholesterol, non-HDL-cholesterol, triacylglycerols

^e^Includes HbA_1c_, fasting glucose, variation of fasting glucose, diabetes duration, type of diabetes, oral hypoglycaemic agent and/or insulin use

^f^Includes history of CVD, ischaemic heart disease, congestive heart failure, CHD or stroke, prior coronary artery bypass graft

^g^Atrial fibrillation, ECG left ventricular hypertrophy, pulse pressure, heart rate, internal carotid IMT, peripheral vascular disease, ABI, cardiac conditions

^h^Renal insufficiency, renal disease, eGFR, micro/macroalbuminuria, uric acid, glutamic pyruvic aminotransferase (GPT), serum creatinine, urine albumin/creatinine ratio, albumin, creatinine clearance

^i^Includes amputation history, ulcer history, neuropathy, absence of monofilament sensation, absence of pedal pulse

^j^Includes retinopathy, history of blindness

^k^Includes white blood cells, haemoglobin, haematocrit, age at completion of formal education, CRP, deprivation score, rheumatoid arthritis, chronic skin infection, uric acid, anticoagulants, fibrinogen factor VII, diet, tinea pedis and/or onychomycosis

ABI, ankle–brachial index; CHF, congestive heart failure; CRP, C-reactive protein; IHD, ischaemic heart disease; IMT, intima–media thickness; MI, myocardial infarction; T1D, type 1 diabetes

### Risk models for the prediction of microvascular complications

#### Retinopathy

Several models have been published for the prediction of different microvascular diseases. Regarding estimation of absolute retinopathy risk, a recent systematic review identified 16 prediction models published by February 2018 [45]. Most of the models were developed in study samples from Europe [31, 46–48], the USA or Canada [14], or a combination of these [49]. The models included some but overall fewer demographic characteristics compared with the CVD scores and most took HbA_1c_ and diabetes duration as predictors into account (Table 3 and electronic supplementary material [ESM Table 1]). External validation and performance comparison in the Diabetes Care System (DCS) cohort, consisting of over 10,000 individuals with type 2 diabetes, showed that the models by Aspelund et al [49], Semeraro et al [48] and Tanaka et al [50] resulted in consistently higher C statistics than the remaining models. These models showed higher discriminatory ability with more severe retinopathy stage (e.g. C statistic [95% CIs] for photocoagulated or proliferative diabetic retinopathy: Aspelund et al 0.89 [0.88, 0.91]; Semeraro et al 0.85 [0.84, 0.87]; and Tanaka et al 0.83 [0.81, 0.85]). Models developed for retinopathy in individuals with type 1 diabetes are sparse. Aspelund et al provided an equation specifically for type 1 diabetes that showed good discrimination in the external validation (C statistic 0.82 [95% CI 0.74, 0.90]) [49, 51].

#### Nephropathy

For the prediction of renal outcomes in individuals with diabetes, several models have been developed, including the RECODe model [14], the UKPDS outcomes model 2 [32], the renal DCS risk score [52] and models developed by Dunkler et al [53] and Jardine et al [54]. Rather than predicting the onset of renal diseases, other models have focused on predicting the progression of chronic kidney disease to kidney failure (e.g. the model developed by Tangri et al [55]). One of the few models predicting end-stage kidney disease in individuals with type 1 diabetes was developed in a cohort from the Steno Diabetes Center Copenhagen and showed very high discrimination in the two performed external validations (C statistic [95% CI]: 0.87 [0.81, 0.92]; and 0.96 [0.94, 0.98]) [56].

#### Neuropathy

A recent systematic review summarised available models predicting polyneuropathy and foot ulcer or amputation as hard endpoints of neuropathy in individuals with diabetes and identified 34 prognostic models [57]. However, most did not allow estimation of absolute risks, thus limiting risk stratification and interpretation to the relative scale and ruling out the assessment of model calibration. The C statistic (95% CI) of 13 models in the DCS study sample [57] for the composite outcome (including foot ulcer and amputation) ranged from 0.53 (0.51, 0.55) to 0.84 (0.82, 0.86), with the model by Boyko et al [58] reaching the highest. One of the few models developed in individuals with type 1 diabetes to predict neuropathy-related outcomes showed good discriminatory ability in the type 1 diabetes subsample of the external validation cohort. However, due to the small sample size (*n* = 49 with type 1 diabetes, including six cases), the estimate was imprecise (C statistic 0.74 [95% CI 0.55, 0.91]) [59].

### Risk models for the prediction of all-cause mortality

Several models have been developed to predict all-cause mortality as the ultimate complication of diabetes. Models that have been externally validated include the RECODe model, the model by Chang et al and the ENFORCE model [14, 60, 61]. The included predictors were mainly demographic, BP- or blood lipid-related, or were renal variables (Table 3 and ESM Table 1). All three models showed acceptable to good discrimination in the external validations, with C statistics of 0.71–0.81 (RECODe), 0.75–0.82 (ENFORCE) and 0.69 (Chang et al) [14, 60–63]. For the prediction of mortality in type 1 diabetes, few models exist and are set mainly in the context of lifetime health outcome simulations [64]. Recently, and equivalently to the UKPDS outcomes model 2, a patient-level simulation model for predicting lifetime health outcomes in type 1 diabetes was developed, including an equation to predict mortality [65]. However, due to the large number of included predictors and the requirement for according information, transferability to the application in clinical routine care is questionable. Overall, the prediction time frames of the identified models for all-cause mortality range between 5 years and 10 years. Particularly for this ultimate complication, longer time horizons may be helpful in order to identify at-risk individuals in a timely manner to enable treatment strategies for risk reduction.

### Risk models for multiple diabetes-related complications and future research directions

It is worth noting the development of different models within single studies predicting multiple diabetes-related complications, including macro- and microvascular complications and/or overall mortality, namely the RECODe models [14], the UKPDS outcomes models 1 & 2 [31, 32], the models by Tanaka et al [50] and Dagliati et al [46]. For example, the RECODe models for macrovascular complications, retinopathy and neuropathy include similar predictors (Table 3 and ESM Table 1). Overlap in the predictor sets may facilitate simultaneous risk assessment of multiple vascular diabetic complications in clinical practice.

Overall, a wide variety of models applicable in clinical practice for the prediction of microvascular complications, and in particular macrovascular complications, as well as mortality is available. Rather than developing new models, future research should focus on external validation and comparison of existing models in target populations, with the aim of providing information about appropriate model choices and implementation.

## Non-classical biomarkers and omics-based predictors

As already discussed, conventional prediction models for macro- and microvascular complications and mortality include a limited set of clinical characteristics and biomarkers based on their availability in routine care. However, information on biomarkers not routinely collected may also be predictive, although their usefulness depends on the extent to which they provide information for prediction not already provided by established risk factors. Thus, novel predictors not only need to be associated with endpoints but also need to demonstrate improvements in risk prediction as evaluated by discrimination, calibration and reclassification statistics.

**Fig. 3:**
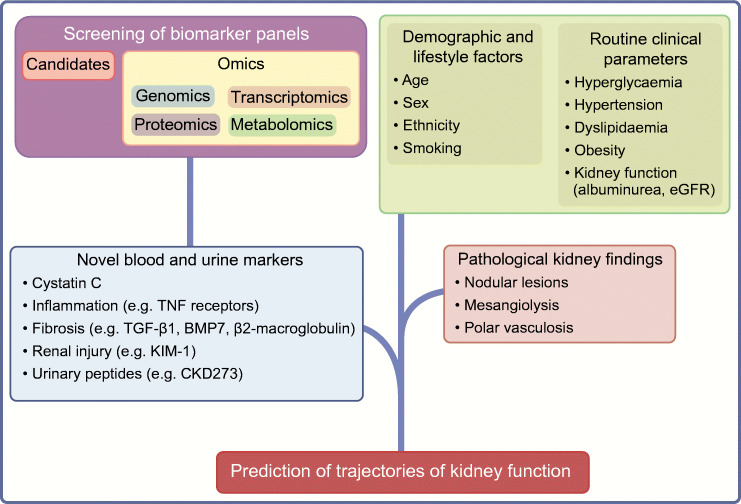
Novel biomarkers for prediction of nephropathy in diabetes. Evaluation of non-conventional blood or urinary biomarkers, either hypothesis-based candidates or from large-scale omics-based technologies, has resulted in several predictive biomarkers for nephropathy. Importantly, such biomarkers need to provide predictive information beyond classical risk factors (demographic and lifestyle factors, routine clinical parameters). BMP7, bone morphogenetic protein 7; KIM-1, kidney injury molecule-1. This figure is available as part of a downloadable slideset

Investigations of predictive biomarkers can either be hypothesis-driven or exploratory. Particularly, methodological developments aimed at identifying, characterising and quantifying biological molecules do now support the screening of high numbers of potentially predictive biomarkers related to the genome, transcriptome, proteome or metabolome (Fig. 3). Numerous studies have investigated individual candidate biomarkers, larger candidate biomarker panels, or omics-based biomarkers and it is beyond the scope of this review to provide a summary of identified biomarkers predictive for different macro- and microvascular complications. Still, such investigations clearly lead to the identification of promising biomarkers with high potential for clinical application.

Figure 3 shows examples of novel biomarkers for prediction of nephropathy in diabetes reviewed elsewhere [66–68]. Screening of individual candidate biomarkers or larger candidate biomarker patterns provides evidence that blood-based markers related to inflammation, fibrosis and renal injury can provide predictive information beyond classical risk factors. For example, circulating levels of TNF receptors and other inflammatory markers have been shown to improve discrimination of future risk of end-stage renal disease compared with clinical markers (albuminuria, eGFR) [69, 70]. Urinary biomarkers also appear to harbour substantial predictive information, beyond the classical markers of kidney function used. Screening of urinary peptides has resulted in a score combining information on 273 peptides (CKD273), having high accuracy in the classification of eGFR status [71]. This score has subsequently been validated to predict rapid progression of eGFR in different cohorts [72].

Specific biomarkers or biomarker combinations can lead to improvements in risk prediction of CVD in diabetes, beyond classical CVD risk factors. As reviewed elsewhere in more detail [73], N-terminal pro B-type natriuretic peptide (NT-proBNP) appears to show particular promise as a risk marker in this context. Still, analyses of larger biomarker panels have revealed a variety of biomarkers that may in combination provide predictive information. For example, screening of 80 circulating proteins measured with a multi-protein assay revealed eight proteins that in combination substantially improved discrimination of major CVD events [74]. Of note, proteins found to predict CVD partly overlap with those implicated for prediction of nephropathy (e.g. TNF receptors, kidney injury molecule [KIM]-1, osteopontin).

Genetic risk scores, combining large numbers of individual gene variants, have been evaluated in recent years in terms of predicting the risk of diabetes complications. In the Action to Control Cardiovascular Risk in Diabetes (ACCORD) and Outcome Reduction With Initial Glargine Intervention (ORIGIN) studies, a polygenetic risk score for coronary artery disease, combining information on 204 variants from genome-wide association studies, had poor discriminative ability for major cardiovascular events (C statistic 0.57) [75]. Still, prediction by clinical risk factors was slightly improved when genetic information was added (AUC difference 0.007, *p*=0.04). Combining genetic risk scores for several complication-related traits to give a multi-polygenetic risk score using a total of ~600 variants yielded moderate discriminative abilities for major macrovascular (C statistic 0.68) and microvascular events (C statistic 0.67) in ADVANCE [76]. This risk score did not outperform a clinical score developed in ADVANCE or the Framingham score for prediction of macrovascular complications, although it did predict CVD and all-cause mortality slightly better than Framingham. Importantly, the risk score included non-genetic information (sex, age at diagnosis, diabetes duration); genetic information alone provided poor discrimination (C statistic for major macro- and microvascular events 0.56). While these results indicate that genetic information does not substantially improve prediction beyond clinical risk factors so far, genetic predictors of complications are of specific interest given that they do not vary during life and may thus be used at diabetes diagnosis or later disease stages without need for reassessment.

Besides classical risk factors and novel biochemical and genetic markers, prediction models for diabetes-related complications have also included other individual characteristics relating to current treatment, comorbidities or the presence of complications other than those predicted [73, 77]. Furthermore, morphological indicators of disease progression may be useful for prediction. For example, examination of kidney biopsies may reveal histopathological changes (e.g. tubular atrophy, nodular lesions) that predict eGFR decline (Fig. 3) [67].

## Towards clinical application of precision prognostic models

For precision prognostics to be successful, it should allow clinicians to match a patient to others with a similar complication risk and optimise therapies for this patient to result in an extended complication-free life. Thus, precision prognostic models are not useful by themselves, but rather they have a positive impact on medical decision making. The availability of validated prognostic models that accurately predict risk is an important first step towards this goal. One major obstacle preventing application of precision prognostic models into care is the largely unknown clinical benefit. Reporting discrimination and calibration will always be important for a prediction model but if the model is to be used for making clinical decisions, decision-analytical measures should be reported. For example, decision-curve analysis, plotting the net benefit of a prognostic model across different threshold probabilities, allows the definition of a single probability threshold that can be used to categorise individuals as positive or negative while weighting false-positive and false-negative classifications [78]. Furthermore, combining prognostic models with potential treatment effects from RCTs may be useful for substantiating the clinical utility of precision prognostics. As an illustration, the Diabetes Lifetime-perspective prediction (DIAL) model [79] allows prospective quantification of future treatment effects on the life-years gained without myocardial infarction or stroke based on clinically available data in individuals with type 2 diabetes. Modelled treatment strategies include smoking cessation, medicinal treatment and therapeutic targets regarding lowering of HbA_1c_ and systolic BP. As a consequence, the model provides information not only on the need for treatment initiation based on the individual risk but also the requirements regarding treatment intensity and combination. Still, very few studies have directly evaluated precision prognostic treatment approaches vs standard care. In the Early detection of diabetic kidney disease by urinary proteomics and subsequent intervention with spironolactone to delay progression (PRIORITY) trial, the urinary proteomic CKD273 score was used to quantify the risk for developing microalbuminuria. Participants who were classified as high risk were entered into an RCT to test whether progression to microalbuminuria could be prevented with the mineralocorticoid receptor antagonist spironolactone. However, development of microalbuminuria was not significantly different from that seen with placebo [68].

Cost-effectiveness analysis should, in addition to treatment effects, be informative for identifying optimal thresholds of predicted risk to target treatments based on precision prediction models, as has been demonstrated for diabetes prevention interventions [80]. In this context, monetary and organisational capacities to collect information beyond those routinely available (e.g. on novel non-routine biomarkers) are likely major obstacles for implementing prognostic models. Cost-effectiveness analyses are important here to prevent the implementation of precision prognostics from leading to reduced access to care and increased rather than reduced health disparities. In addition, there is a risk that more precise prognostication may cause distress if the options for successful intervention are limited or incompatible with an individual’s needs or desires [1].

Statistical models to calculate absolute risks need to be ‘translated’ into test instruments for their practical use. In this context, effective strategies to communicate absolute risks and risk limits or classifications are necessary to enable clinicians and patients to make treatment decisions. As an example, the Joint Asia Diabetes Evaluation platform, a web-based data collection and decision support system, provides personalised risk categorisation and absolute risk estimation for CVD and retinopathy. Individuals with diabetes enrolled in these integrated care programmes experienced lower rates of major complications than those in routine care [81]. However, these individuals were not randomised and care programmes differed by elements other than prognostic models, making it difficult to attribute difference in outcomes to precision prognostics.

Given the largely unknown clinical benefit of precision prognostics, it is no surprise that there is currently limited reference to prognostic models in medical guidelines for the treatment of diabetes. The ADA recommends the use of the Pooled Cohort Equation CVD risk model, although this model was not developed specifically in individuals with diabetes [82]. Still, recommendations for treatment intensity and targets for major atherosclerotic CVD risk factors such as BP and blood lipids are partly based on an assessment of absolute CVD risk. In contrast, the European Society of Cardiology (ESC) and EASD recommend ‘conventional’ CVD risk stratification, based on the presence of prevalent CVD and CVD risk factors but without inclusion of a prognostic model [83]. Interestingly, the ESC/EASD specifically discourage the use of risk prediction models developed for the general population in individuals with diabetes [82]. With regard to microvascular complications, current ADA guidelines [84] do not consider the use of risk prediction models. Thus, despite the existence of several validated models for prediction of macro- and microvascular complications in individuals with diabetes, their application in routine care is currently not encouraged.

## Outlook

Although an increasing number of prognostic models have been developed and validated to predict diabetes complications, the concept of precision prognosis as a component of precision medicine is still in its infancy. Epidemiological and clinical research could inform its further development (Text box 2). 
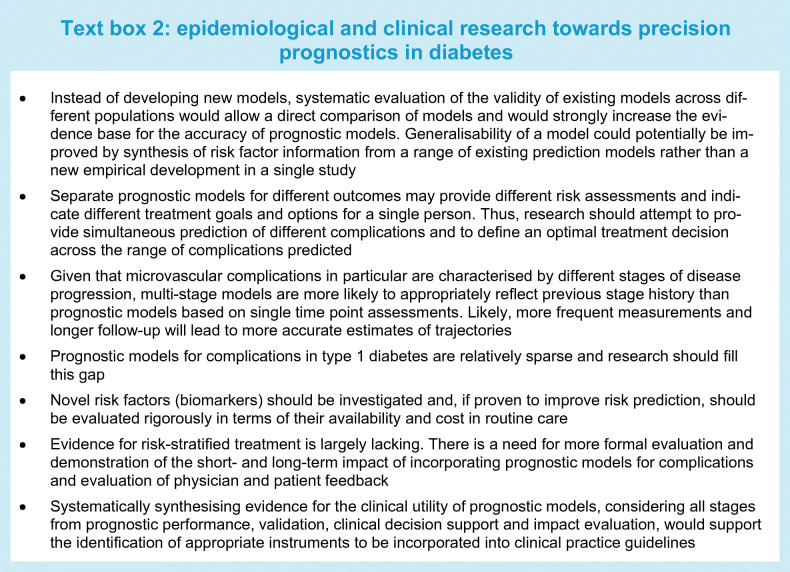


## Supplementary information

ESM Table 1 (XLSX 39 kb)

Slideset of figures (PPTX 329 kb)

## Abbreviations

ADVANCE: Action in Diabetes and Vascular Disease: Preterax and Diamicron MR Controlled Evaluation
DCS: Diabetes Care System
EPIC: European Prospective Investigation into Cancer and Nutrition
ESC: European Society of Cardiology
NDR: National Diabetes Register
RECODe: Risk Equations for Complications of Type 2 Diabetes
UKPDS: UK Prospective Diabetes Study
