# Supplementary material for: Precision prognostics for the development of complications in diabetes
Source: Diabetologia. 2022 Jun 21;65(11):1867–82. doi: 10.1007/s00125-022-05731-4 (PMC9522742; doi:10.1007/s00125-022-05731-4)
Supplement: Supplementary file 2 — (PPTX 329 kb) [file 125_2022_5731_MOESM2_ESM.pptx]

## Slide 1
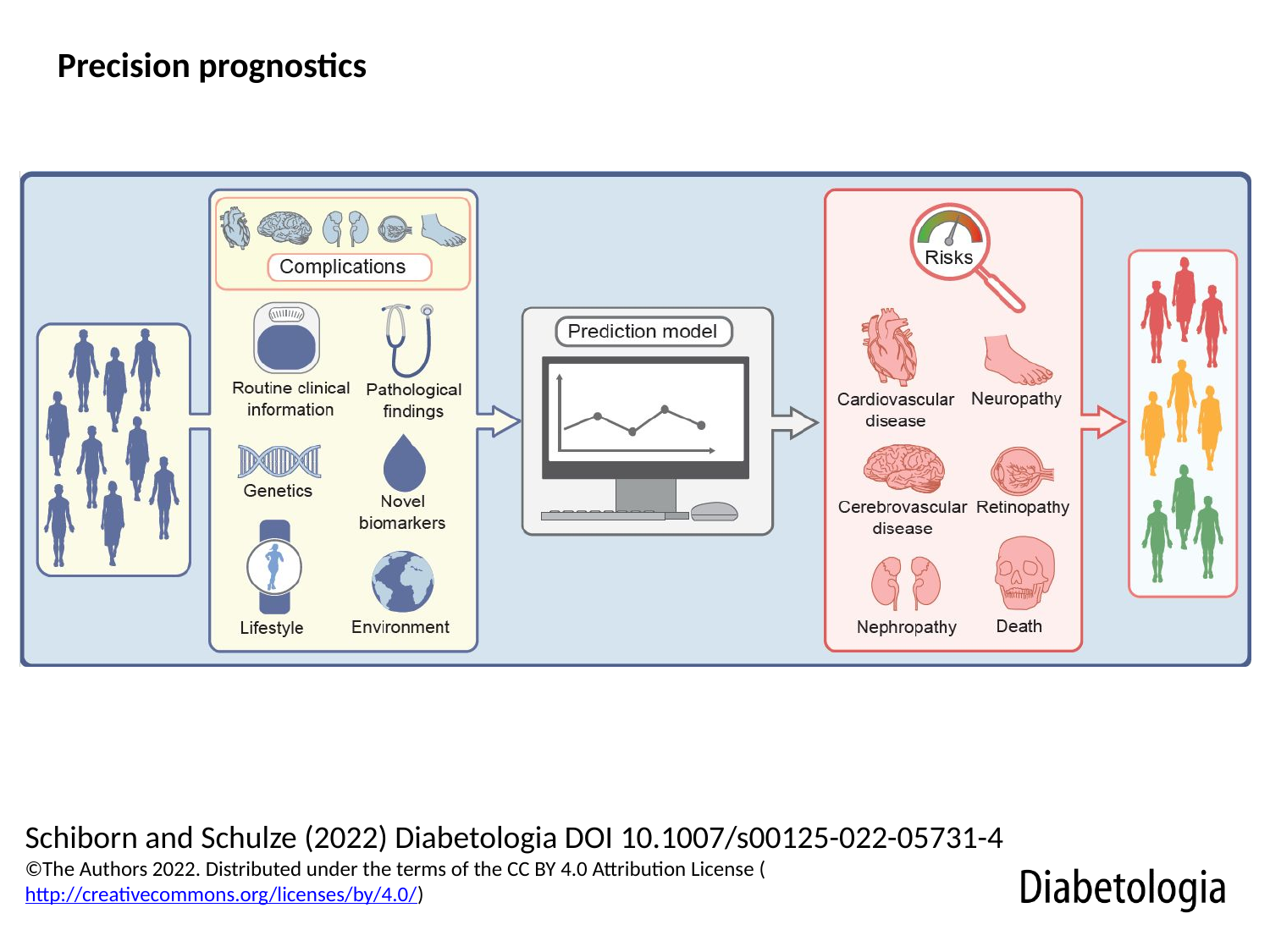

Precision prognostics
Schiborn and Schulze (2022) Diabetologia DOI 10.1007/s00125-022-05731-4
©The Authors 2022. Distributed under the terms of the CC BY 4.0 Attribution License (http://creativecommons.org/licenses/by/4.0/)

## Slide 2
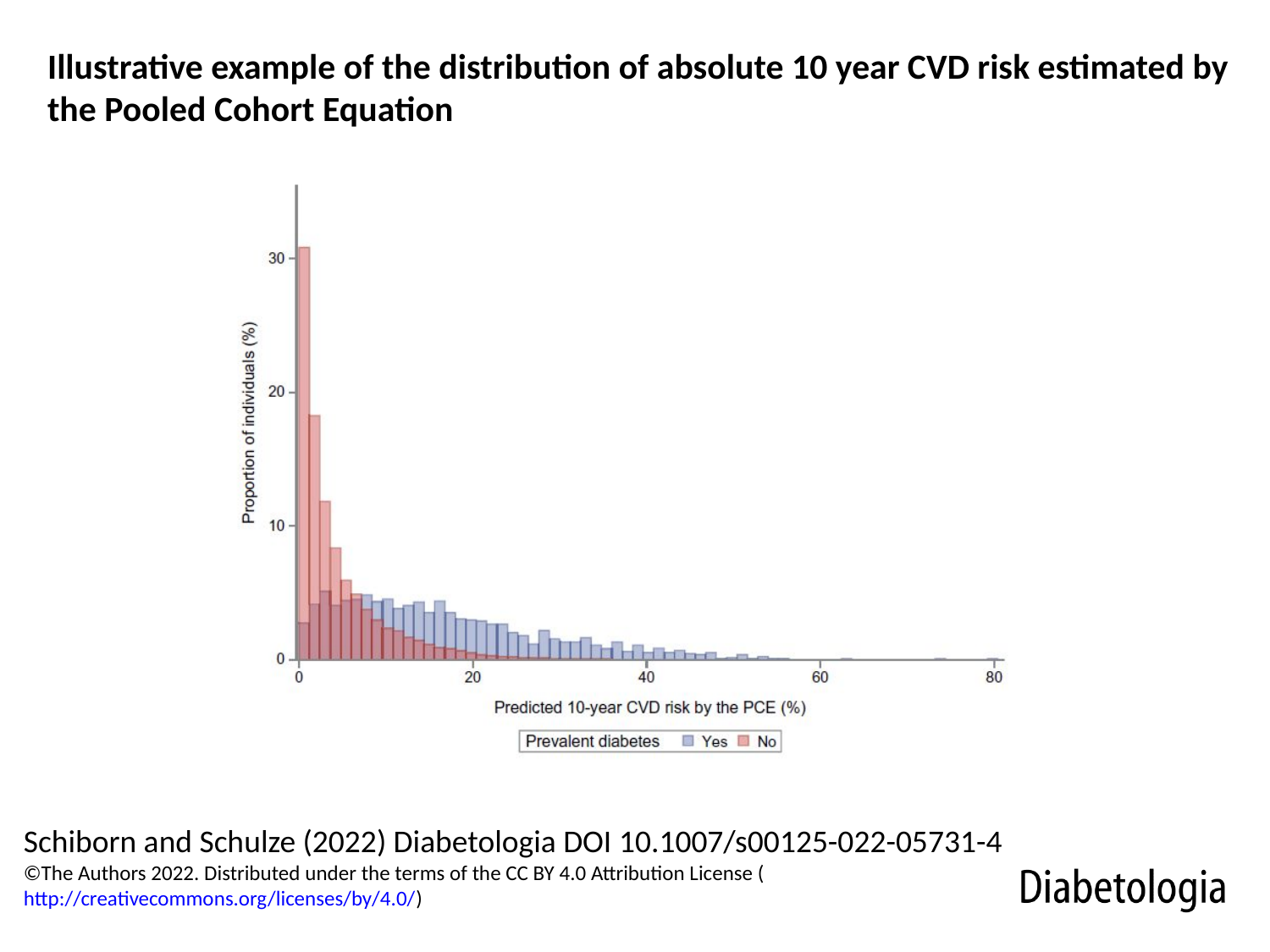

Illustrative example of the distribution of absolute 10 year CVD risk estimated by the Pooled Cohort Equation
Schiborn and Schulze (2022) Diabetologia DOI 10.1007/s00125-022-05731-4
©The Authors 2022. Distributed under the terms of the CC BY 4.0 Attribution License (http://creativecommons.org/licenses/by/4.0/)

## Slide 3
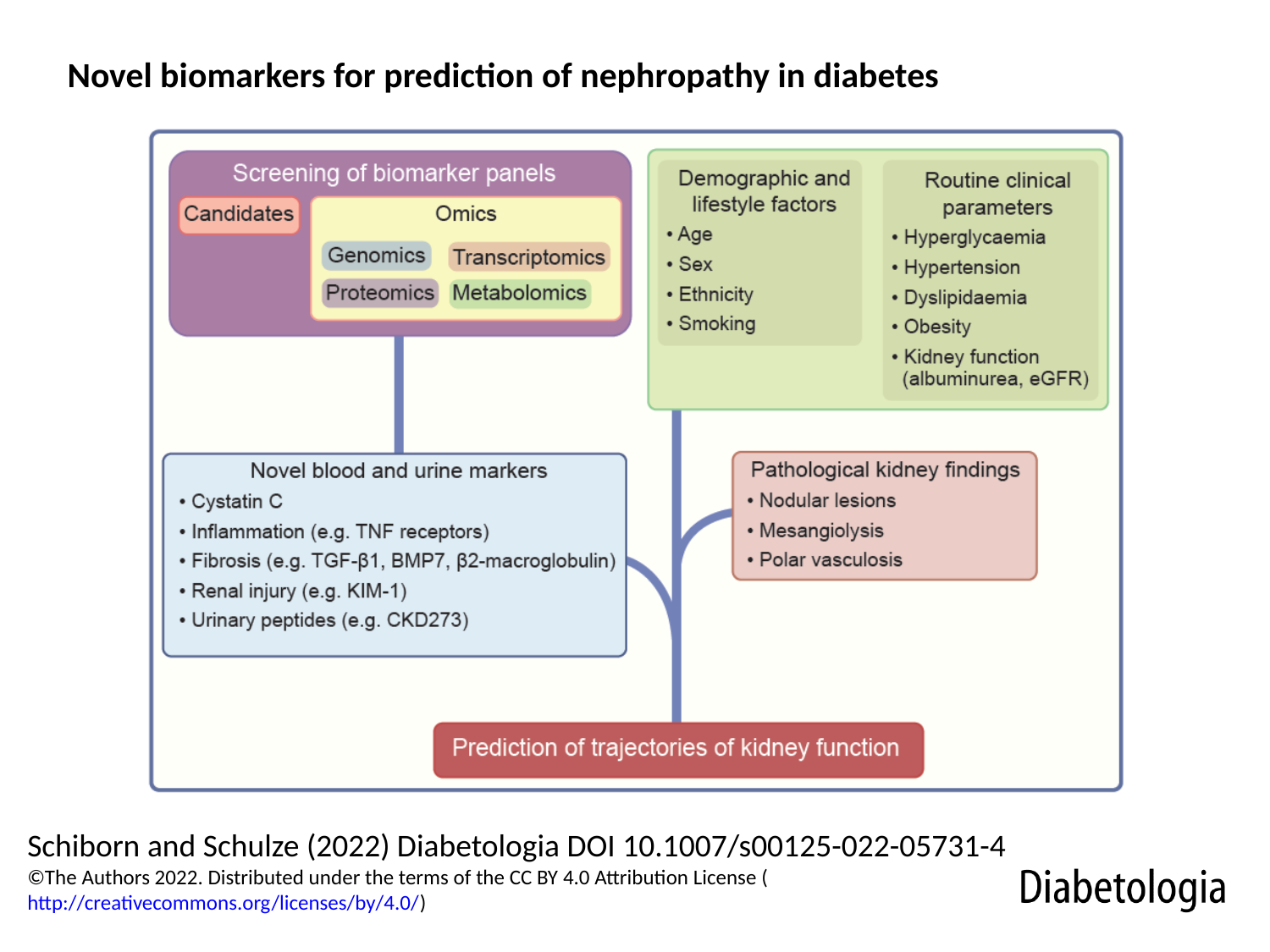

Novel biomarkers for prediction of nephropathy in diabetes
Schiborn and Schulze (2022) Diabetologia DOI 10.1007/s00125-022-05731-4
©The Authors 2022. Distributed under the terms of the CC BY 4.0 Attribution License (http://creativecommons.org/licenses/by/4.0/)
